# Potential Application of Essential Oils for Mitigation of *Listeria monocytogenes* in Meat and Poultry Products

**DOI:** 10.3389/fnut.2020.577287

**Published:** 2020-11-24

**Authors:** Mojtaba Yousefi, Nasim Khorshidian, Hedayat Hosseini

**Affiliations:** ^1^Food Safety Research Center (Salt), Semnan University of Medical Sciences, Semnan, Iran; ^2^Department of Food Science and Technology, Faculty of Nutrition Sciences and Food Technology, National Nutrition and Food Technology Research Institute, Shahid Beheshti University of Medical Sciences, Tehran, Iran

**Keywords:** meat, essential oil, preservatives, natural, antimicrobial, *Listeria monocytogenes*

## Abstract

One of the most important challenges in the food industry is to provide healthy and safe food. Therefore, it is not possible to achieve this without different processes and the use of various additives. In order to improve safety and extend the shelf life of food products, various synthetic preservatives have been widely utilized by the food industry to prevent growth of spoilage and pathogenic microorganisms. On the other hand, consumers' preference to consume food products with natural additives induced food industries to use natural-based preservatives in their production. It has been observed that herbal extracts and their essential oils could be potentially considered as a replacement for chemical antimicrobials. Antimicrobial properties of plant essential oils are derived from some main bioactive components such as phenolic acids, terpenes, aldehydes, and flavonoids that are present in essential oils. Various mechanisms such as changing the fatty acid profile and structure of cell membranes and increasing the cell permeability as well as affecting membrane proteins and inhibition of functional properties of the cell wall are effective in antimicrobial activity of essential oils. Therefore, our objective is to revise the effect of various essential oils and their bioactive components against *Listeria monocytogenes* in meat and poultry products.

## Introduction

Food safety is one of the most important issues in the food industry. In fact, concerns about pathogenic microbes causing foodborne diseases are manifested by consumers, food manufacturers, and regulatory organizations (1, 2). Therefore, the food industry wants to produce high-quality and safe foodstuff (3, 4). Hence, part of the research activities has always been dedicated to increasing knowledge about the production of safe food and the development of new methods applied to improve their safety.

One of the foodstuff that must be safely produced and stored under hygienic conditions is meat and meat products. These are rich in essential nutrients and extremely prone to microbial and chemical deterioration if not well processed and preserved. Therefore, poor hygienic conditions of processing and storage lead to microbial contamination, which can lead to safety and spoilage problems (4–7).

Various microorganisms such as bacteria, mold, and yeast are involved in the spoilage of meat and meat products. Furthermore, inappropriate production and storage condition of meat and meat products lead to incidence of diseases, which is caused by various pathogens such as *Clostridium* spp., *Salmonella* spp., *Campylobacter jejuni, Escherichia coli*, O157:H7, *Aeromonas hydrophila*, and *Listeria monocytogenes*, among which, *L. monocytogenes* is considered as the major causative agent responsible for serious diseases in both humans and animals (8, 9).

*L. monocytogenes* is frequently isolated in various food products. This pathogen results in listeriosis, which remarkably affects pregnant women, newborns, and individuals with immunodeficiency (10, 11). Due to the ability of this microorganism to growth at low temperatures (2–4°C), there is a particular concern about the presence of *L. monocytogenes* in meat and poultry products (12, 13). Various thermal and non-thermal methods including heat treatment, high hydrostatic pressure, irradiation, and high-intensity pulsed electric field processing as well as different methods of packaging have been utilized to produce safe food (11, 14–17).

Also, various preservatives have been utilized to hinder contamination during production, distribution, and storage as well as to increase shelf life of raw and processed meat and poultry products. Although food-grade and Generally Recognized as Safe (GRAS) synthetic additives have been usually used in the food industry, in recent years, consumers have shown an increasing concern about the use of synthetic chemical preservatives (18–20). Therefore, there is an increasing tendency in using natural additives including antioxidants, antimicrobials, sweeteners, and coloring agents that originated from animals, plants, and microorganisms (21, 22). Various naturally occurring antimicrobial agents have been recognized. Lactoperoxidase, lactoferrin, lysozyme from animal sources, bacteriocins and natamycin from microbial sources, and essential oils (EOs) from plant sources are examples of natural preservatives (3, 21). EOs that are volatile and lipophilic liquids, obtained from diverse plant organs such as seeds, roots, stems, buds, flowers, and wood, exert antimicrobial and antioxidant properties (23–25). Various studies have reported that EOs from aromatic and medicinal plants have antimicrobial properties against *L. monocytogenes* (4, 20, 26–31). Due to the described antimicrobial activity of EOs against various microorganisms, this study aimed to review the effect of various EOs on *L. monocytogenes* when applied to meat and poultry products.

## *L. monocytogenes* in Meat and Meat Products

The presence of *Listeria* spp. in meat and meat products is a serious problem in the meat industry due to the ability of this organism to grow in both raw and cooked meat during refrigerated storage, and among the food products, contaminated meat products are known to be one of the main sources for *L. monocytogenes* infections (32–34).

*L. monocytogenes* is a pathogenic, Gram-positive, non-spore-forming, facultative anaerobic, highly mobile, rod-shaped bacterium (35–37). *L. monocytogenes* is a major causative agent of foodborne illness worldwide. The severe invasive disease caused by *L. monocytogenes* is listeriosis (38). It has been indicated that listeriosis carries high rates of hospitalization and mortality. Nearly 94% of confirmed cases of listeriosis need to be hospitalized, and 14% of them die (9, 39). Based on the somatic O antigen, *L. monocytogenes* can be subclassified into 13 serotypes. All the 13 serotypes can cause listeriosis; however, serotypes 1.2b, 1.2a, and 4b are more widespread (27, 40). Apart from food matrix diversity, capacity for pathogenicity, and geographical area, the most isolated serotypes from food products are 4c, 4b, 3b, 1.2a, and 1.2b (9, 41).

*L. monocytogenes* is a psychrotrophic bacterium and can grow over a wide range of temperatures (1–45°C) and pHs (4.3–9.4) and at water activity with a value of 0.92 and above (36, 42). In comparison to other foodborne pathogens, *L. monocytogenes* can tolerate undesirable environmental conditions such as low-oxygen conditions, nitrite, and high salt content. Furthermore, it can persist in the environment, processing plants, and food products at refrigerated temperatures for a long time (43, 44). The ability of *L. monocytogenes* in forming biofilms allows it to remain successfully in food processing establishments and retails (45). Due to the formation of biofilms and attachment of *L. monocytogenes* to various surfaces in food establishments, it is hard to eradicate this pathogen without the performance of precise sanitary protocols. Indeed, because biocides are often highly chemically reactive molecules, the presence of various organic compounds such as polysaccharides, nucleic acids, and proteins can remarkably weaken their efficiency. Furthermore, possible interactions between antimicrobials and biofilm components might explain the limitations of penetration into the biofilm (36, 44, 46).

Due to the ability of *L. monocytogenes* to survive and grow in dry, cold, and high-salt environments, it is widely distributed in different matrices such soil, water, and various food products including meat, fish products, vegetables, dairy products, and ready-to-eat (RTE) food (9, 47).

Since many listeriosis outbreaks have been linked to meat product consumption, prevention of meat and meat product contamination with *L. monocytogenes* is one of the major concerns of the meat processing industry (40). Thermal processing of meat products can easily eliminate *L. monocytogenes*. However, posterior contamination of meat products especially RTE meat products with this pathogen is frequent. Various post-package decontamination strategies such as in-package thermal pasteurization, irradiation, high-pressure processing, and use of antimicrobial additives in the formulation of meat products have been utilized to mitigate and control the growth of *L. monocytogenes* in meat and poultry products (48). Various food-grade synthetic preservatives and antimicrobial agents have been utilized to prevent the growth of *L. monocytogenes*; however, due to increasing awareness of consumers about the potential adverse effects of synthetic preservatives, more researches have been developed to determine the potential use of natural additives and antimicrobial compounds in the food industry. Among these, there has been great attention in using EOs as natural antimicrobials and antioxidants in the formulation of meat and poultry products.

## EOs Composition and Mechanisms of Their Antimicrobial Activity

As aforementioned, herbal extracts and EOs from plants can be considered as potential alternatives to artificial preservatives to improve the shelf life and the safety of food products such as meat and poultry and RTE meat products (49). EOs, which also known as volatile or ethereal oils, are naturally aromatic components found in many plants. They can be obtained from various parts of plants including buds, flowers, seeds, leaves, roots, peels, fruits, barks, and woods through only physical extraction and isolation such as pressing and distillation (3, 49).

EOs are made of different compounds characterized by colorless to slightly yellowish liquid and poorly soluble or insoluble in water, but soluble in organic solvents (50). EOs mostly possess a pleasant odor and sometimes a specific taste, and they are utilized in considerable amount in the flavoring and perfume industries. Various fragrance extraction methods including cold pressing and extraction and distillation such as steam distillation are used in order to prepare and obtain EOs (51–53). In total, almost 3,000 EOs are known, among which 300 are commercially utilized in pharmaceutical, food, agronomic, sanitary, cosmetic, and perfume industries (54). The species of plant, plant geographic origin, climate, composition of soil, the vegetative stage of the plant, and the part of plant that is utilized for extraction of EO are the factors that affect the composition of EOs (55–57). EOs are usually secreted as secondary metabolites which exhibit antibacterial, antifungal, and antibiofilm properties (58). These various biological activities are directly related to the bioactive volatile components that are present in EOs (25, 52). Almost 90–95% of EOs are volatile components such as aliphatic aldehydes, alcohols, esters, monoterpenes, and sesquiterpene hydrocarbons and their oxygenated derivatives. The nonvolatile part, which makes up 5–10% of EOs, comprises hydrocarbons, fatty acids, sterols, carotenoids, waxes, coumarins, and flavonoids (3, 59).

Apart from the fact that EOs are mainly used as flavoring agents in the food industry, due to their antimicrobial properties, they can also be used in foodstuff to increase shelf life. The main drawback for the application of EOs as antimicrobial agents is the creation of strong aromas and off-flavors, limiting the use of high concentrations (44, 52). Therefore, it is essential to have information about the target microorganisms, properties of EOs, minimum inhibitory concentrations (MICs), mechanisms of action of EOs, and their interaction with the matrix and sensory properties of the food (60). The antimicrobial properties of EOs are ascribed to the action of various compounds that can be generally divided into terpene and phenolic compounds (44).

As aforementioned, EOs have antimicrobial activities against a wide range of microorganisms; however, the exact antimicrobial mechanism has not been completely elucidated, and it cannot be attributed to an individual mechanism. It seems that based on the chemical compounds contained in the EOs, several mechanisms are involved in the antimicrobial properties of EOs (50, 52). It has been mentioned that the antimicrobial activity of EOs may be due to the possible penetration of EOs through the bacterial cell wall and exertion of inhibitory effects on the functional properties of the cell (61, 62). The hydrophobicity of EOs lets them break down the lipid layer of the bacterial cell membrane and mitochondrion, making the structure more penetrable, and therefore, leakage of ions and other cell compounds occurs, and when the leakages are more than the limit, cell death occurs (52, 63). It has been indicated that disruption of the cell membrane by phenolic compounds of EOs causes exudation of the internal contents of the cell and inhibits functional properties of the cell (49). It has been proposed that phenolic compounds of EOs exert antimicrobial properties by changing the permeability of the microbial cell, damaging cytoplasmic membranes, intervening in the generation system of cellular energy (ATP), and disrupting the proton motive force (49, 53, 64, 65).

Generally, the interaction of EOs with cell membranes of bacteria can be effective in preventing bacterial growth. The hydrophilic or lipophilic properties of EO constituents, type of microorganism, and structure of the cell wall are the factors that affect the antimicrobial activity of EOs (52, 60, 66). Furthermore, the shape of the bacteria can be effective in EO activity, and it has been indicated that rod-shaped cells are more sensitive to EOs in comparison with coccoid-shaped cells (50). It is indicated that Gram-positive bacteria are more sensitive to EOs in comparison with Gram-negative ones.

**Figure 1:**
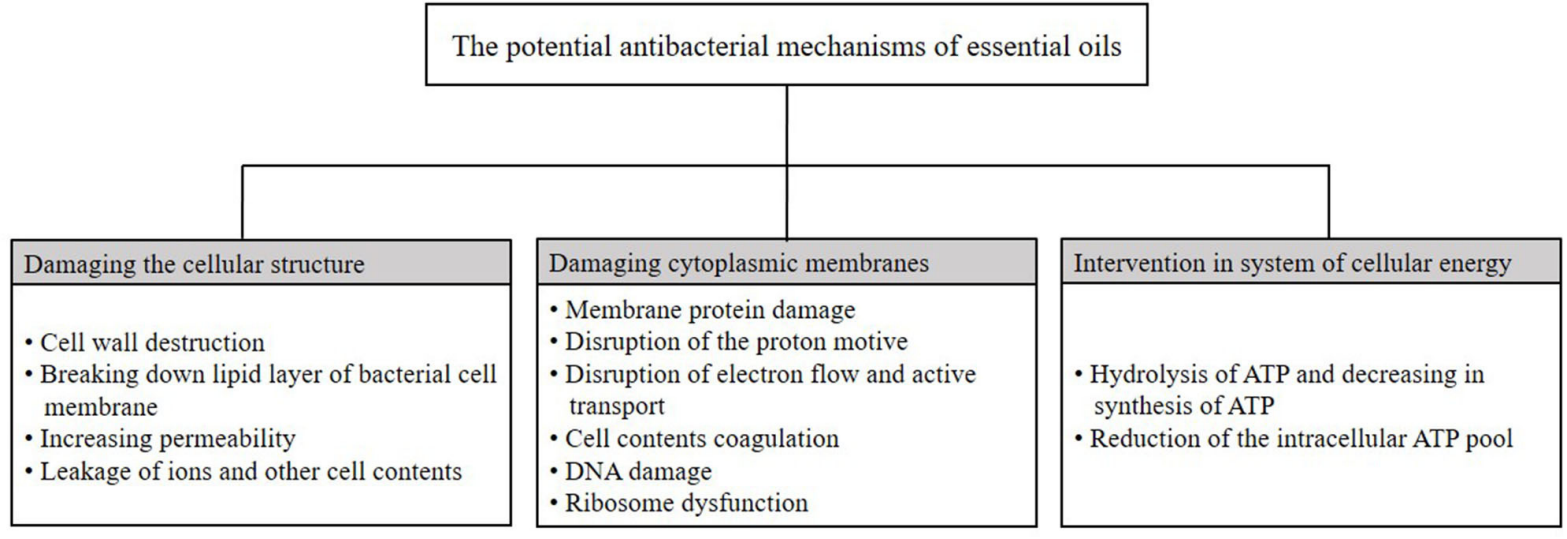
The potential mechanisms of essential oils activity on microorganisms.

It seems that the sensitivity of Gram-positive bacteria is related to the direct interaction of the hydrophobic components of the EOs with the cell wall (66–68). The cell wall of Gram-positive bacteria is made of a thick layer of peptidoglycans (90–95%), teichoic acid, and proteins (44, 69). Due to the hydrophobic nature of major parts of EOs, they can easily pass through it. On the other hand, Gram-negative bacteria have a more complex structure including a monolayer of peptidoglycans surrounded by an outer layer comprising proteins and lipopolysaccharides (LPS). This outer cell membrane is charged and possesses a hydrophilic nature, and therefore, diffusion of hydrophobic compound is limited through LPS (3, 50, 70). Therefore, due to structure variation in the outer layers of bacteria, Gram-positive bacteria such as *Staphylococcus aureus, Bacillus cereus*, and *L. monocytogenes* can be more easily inhibited by EOs than Gram-negative bacteria such as *E. coli* and *Salmonella enteritidis* (52, 53). The possible basic mechanisms of EO antimicrobial activity are shown in Figure 1.

## Application of EOs in Meat and Poultry Products

As aforementioned, there has been a growing interest in replacing synthetics additives with natural antioxidants and antimicrobials. EOs are one of these natural additives that have been utilized as antioxidants, antimicrobials, and flavoring agents in meat and poultry products. As Gram-positive organisms are more susceptible to EOs, attention has been focused on utilizing EOs in the inhibition of Gram-positive bacteria such as *L. monocytogenes* in meat and poultry products.

Upadhyay et al. studied the effect of GRAS plant-derived antimicrobial compounds against *L. monocytogenes* in frankfurters by applying them as post-processing dip treatments. The surface of frankfurters was inoculated with a mixture of five strains of *L. monocytogenes* (~6.0 log CFU per frankfurter) and treated at 55°C (60 s) and 65°C (30 s) in in sterile deionized water or water with as β-resorcylic acid (1.5%), carvacrol (0.75%), and *trans*-cinnamaldehyde (0.75%) alone. After that, the samples were vacuum-packaged (VP) and kept at 4°C for 70 days. They found that the application of plant-derived compounds as antimicrobial dips was effective in preventing growth of *L. monocytogenes* on frankfurters during refrigerated storage. They also found that β-resorcylic acid had the highest activity against *L. monocytogenes* in comparison to other individual antimicrobial treatments. They concluded that plant-derived antimicrobial compounds could be efficiently utilized as post-processing dips to decrease *L. monocytogenes* on frankfurters (71). The antimicrobial activities of *Thymus capitata* EO against *L. monocytogenes* ATCC 19118 inoculated in minced beef meat were investigated by El Abed et al. They also investigated anti-*Listeria* activity of various concentrations [0.01, 0.05, 0.25, and 1.25% (v/w)] of *T. capitata* EO in minced beef meat and found that by increasing EO concentration, a gradual decrease in *L. monocytogenes* ATCC 19118 count occurred. They figured out that the *L. monocytogenes* population was significantly decreased by the application of 0.25 or 1.25% (v/w) of *T. capitata* EO to minced beef in comparison to control samples (18). Moon et al. studied the synergism effect of soy sauce and teriyaki sauce with carvacrol or thymol (0.3 and 0.5%) as common natural compounds in controlling *L. monocytogenes* in marinated beef stored at 4°C for 7 days. They figured out that *L. monocytogenes* was not inhibited by usage of Teriyaki sauce alone, while teriyaki sauce in combination with 0.5% carvacrol or thymol inactivated *L. monocytogenes* during 7 days of storage (72).

Giarratana et al. studied the effect of thyme and rosemary EOs (0.025 and 0.05%) against a mix of three strains of *L. monocytogenes* (*L. monocytogenes* ATCC 19111, ATCC 13932, and ATCC 19117) in Italian mortadella packaged in a modified atmosphere and kept at 4°C for 30 days. Their results revealed that the mixture of rosemary and thyme EOs had a bacteriostatic activity against *L. monocytogenes* and that both 0.025 and 0.05% of tested EOs significantly inhibited *L. monocytogenes* growth compared with the control sample. The *L. monocytogenes* population increased from approximately 2.50 log CFU/g to 5.31, 3.01, and 2.52 log CFU/g in control, 0.025% EO-treated, and 0.05% EO-treated samples, respectively. They indicated that heat treatment of mortadella at 80°C for 4 h could change the antimicrobial activity of tested EOs. Therefore, complementary preservation strategies such as modified atmosphere packaging (MAP) can improve the EO antimicrobial effect. They also studied the effect of lactic acid bacteria (LAB) growth and pH changes of mortadella on antimicrobial activity of tested EOs and understood that with the growth of LAB and decrease of pH values in mortadella, bacteriostatic activity of tested EOs against as *L. monocytogenes* increased (12). It has been indicated that the pH of the food matrix affects the activity of EOs and that the hydrophobicity of some EOs increased at low pH. Therefore, EOs can more easily penetrate the lipid part of the bacterial membrane and hence exert increased antimicrobial activities (12, 53, 73). Similarly, Gouveia et al. evaluated the antimicrobial effect of rosemary (*Rosmarinus officinalis* L.) and thyme (*Thymus vulgaris* L.) against *L. monocytogenes* ATCC 679 in sous vide cook–chill beef at 2 and 8°C during 28 days of storage. Their results showed that thyme and rosemary EOs at 3.9 and 62.5 μl/ml could inhibit *L. monocytogenes*. They also studied the effect of EOs at MIC values on the inhibition of *L. monocytogenes* in beef samples. The sample containing thyme EOs and the control sample had similar counts of *L. monocytogenes*. On the other hand, a 2-log CFU/g reduction occurred in the rosemary-treated samples stored at 2 and 8°C. They concluded that rosemary EO can be potentially used as a natural preservative. They indicated that the lower antimicrobial effect of thyme EOs could be attributed to the low concentrations of thymol (phenolic compound) in the *T. vulgaris* chemotype that was utilized in their investigation (74). Conversely, a higher antimicrobial activity of thyme EO against *L. monocytogenes* in minced meat stored at 4°C was reported by Pesavento et al. They stated that *p*-cymene (47.9%) and thymol (43.1%) were the main antimicrobial constituents (75). In contrast, in the study carried out by Gouveia et al., the reduced antimicrobial activity of tested thymol EOs was associated with lower levels of *p*-cymene (4.91%) and thymol (7.48%). They also indicated that the decreased antimicrobial activity of thymol EO can be associated with the lower concentrations used (74). It is stated that *p*-cymene can be placed on the bacterial membrane and interfere with its function. Furthermore, the thymol compound, which is a phenolic monoterpenoid, has a phenolic ring and can cause functional and structural damage to the bacterial cytoplasmic membrane (60).

In a study by Mytle et al., the antimicrobial activity of clove (*Syzygium aromaticum*) EOs (1 and 2%, v/w) on RTE chicken frankfurters, which were inoculated with seven strains of *L. monocytogenes* (10^2^-10^6^ CFU/g) and stored for 14 days (at 5 or 15°C), was determined. They found that all the tested strains were able to survive and grow in the control sample at 5 and 15°C, while the addition of either 1 or 2% clove EO inhibited bacterial growth under both storage conditions. They indicated that clove EO (1% v/w) along with low-temperature storage could decrease possible contamination and growth of *L. monocytogenes* without having an adverse effect on flavor (76). Furthermore, Khaleque et al. studied the application of clove and cinnamon EO against *L. monocytogenes* in ground beef. They studied the effect of 5 and 10% of crude and commercial clove EO or 2.5 and 5.0% of crude and commercial cinnamon EO against *L. monocytogenes* in ground beef stored at 0 and 8°C for 7 days and at −18°C for 60 days. They realized that 10% of either crude or commercial clove EOs were able to entirely inhibit *L. monocytogenes* in ground beef 3 days post inoculation, regardless of storage temperature, while either crude or commercial clove EOs at 5% concentration was not able to effectively inactivate *L. monocytogenes* during storage. Furthermore, both 2.5 and 5.0% cinnamon EOs were not able to kill *L. monocytogenes* during storage. On the other hand, based on the storage time and temperature, a decrease of 3.5–4.0 log CFU/g in the *L. monocytogenes* population occurred with the addition of 5.0% commercial cinnamon EOs. This bacterial count reduction was achieved after 7 days of refrigeration and chilling temperatures and 60 days of freezing temperatures, indicating that anti-*Listeria* activity of cinnamon EO is affected by time and temperature. However anti-*Listeria* activity of clove EO was not affected by time and temperature, and therefore, clove EO can be more effective in the inactivation of *L. monocytogenes* in ground beef than cinnamon (4). Similarly, it has been reported by various investigations that clove and cinnamon EOs could be effective in the inhibition of *L. monocytogenes* and in expanding the shelf life of meat (77, 78). The antibacterial effect of clove is related to eugenol, a member of the phenylpropanoid class of compounds that cause the deterioration of the cell wall and lysis of bacterial cell (70). Furthermore, the antibacterial activity of cinnamon EOs is derived from compounds such as cinnamaldehyde, limonene, and eugenol (52). It was stated that eugenol could change the membrane and fatty acid profile, affect the transportation of ATP and ion, and inhibit ATPase, histidine decarboxylase, amylase, and protease enzymes (50, 79).

Additionally, in a study carried out by Raeisi et al., the effects of sodium alginate coating with nisin, cinnamon, and rosemary EOs individually and in combinations on the fate of *L. monocytogenes* in chicken meat during 15 days of refrigeration were studied. The control and the sample coated with alginate solution had the highest growth rate of *L. monocytogenes*, while other treated samples, especially those with the combined use of tested antimicrobial agents, resulted in the inhibition of *L. monocytogenes*, whereas the combination of cinnamon and rosemary EOs, rosemary EOs and nisin, and cinnamon EOs and nisin had the lowest final population, respectively, indicating the synergistic effect of these EOs and nisin in controlling *L. monocytogenes* (80). It seems that cinnamaldehyde and camphor as the main components of cinnamon and rosemary EOs contribute to their antibacterial activities by disrupting the function of the cytoplasmic membrane, electron flow, proton motive force, and coagulation of cell contents (53). It has been reported by Tajik et al. that *L. monocytogenes* was more inhibited by the simultaneous use of *Zataria multiflora* EO and grape seed extract in comparison with individual use of *Z. multiflora* EO in buffalo patties (81). Based on the type of antimicrobial agents and microorganisms, the combined effect of different antimicrobial compounds might be additive, antagonistic, or synergistic (82).

Firouzi et al. studied the effect of oregano and nutmeg EOs (1, 2, and 3 μl/g) on *L. monocytogenes* in ready-to-cook Iranian barbecued chicken that was inoculated with 6–7 log CFU/g of this pathogen and stored at 3, 8, and 20°C for 72 h. They reported that nutmeg with a MIC value of 0.20 μl/ml was more effective against *L. monocytogenes* than oregano EO with a MIC value of 0.26 μl/ml. Furthermore, MBC values of 0.50 and 0.54 μl/ml were obtained against *L. monocytogenes* by nutmeg and oregano EOs, respectively. They also found that there were no significant differences among all EO-treated and control Iranian barbecued chickens in *L. monocytogenes* growth during 72 h of storage at any of the three temperatures (3, 8, and 20°C) (83). Furthermore, the effect of *N,O*-carboxymethyl chitosan, oregano EO, and their combination on *L. monocytogenes* in raw chicken meat filets that were inoculated with low (10^3^ CFU/g) and high (10^5^ CFU/g) counts of bacteria and stored at 4°C for 14 days was studied by Khanjari et al. Their results showed that *N,O*-carboxymethyl chitosan exerted a significantly stronger antimicrobial activity against *L. monocytogenes* when compared to oregano EO. They found that *L. monocytogenes* was completely inhibited by the combination of *N,O*-carboxymethyl chitosan and oregano EO in the samples with low and high inoculation levels at days of 2 and 4 of storage, respectively (84). Similarly, Pavli et al. found that incorporation of oregano EO into sodium alginate edible films in ham slices led to a 1.5-log CFU/g decrease in population of *L. monocytogenes* at the end of the storage (40 days) at 8 and 12°C and an approximately 2.5-log CFU/g reduction at 4°C. They finally indicated that a significant reduction or absence of *L. monocytogenes* was achieved in ham slices by application of high hydrostatic pressure and edible film containing oregano EO, together (85). Additionally, the combination effect of packaging atmosphere, oregano EO, and cold temperature on inhibition of *L. monocytogenes* Scott A in RTE smoked turkey meat was studied by Mahgoub et al. They found that inhibition of *L. monocytogenes* in RTE smoked turkey under MAP and MAP with oregano EO (MAPEO) was increased when compared to VP during the shelf life of the product. They stated that *L. monocytogenes* can be efficiently controlled by a combination of MAP and oregano EO, especially when it is difficult to keep constant temperature during transportation and retail display (86). The antimicrobial activity of oregano EO has been previously confirmed, and it has been utilized to control *L. monocytogenes* in meat and meat products (75, 87–89). The composition of oregano EO is a mixture of volatile terpenes, including α-terpinene, *p*-cymene, carvacrol, and thymol, that participate in antimicrobial properties of this EO, and the latter two compounds are the most important due to their activities on the membranes of bacteria (90). Furthermore, it has been reported that *Z. multiflora* Boiss EO, which mainly contains thymol and carvacrol, had antimicrobial activities against *L. monocytogenes* in meat and meat products, indicating the importance of these two components in the antimicrobial activities of the EOs (31, 91).

It has been stated that the outer membrane of microorganisms was disintegrated by thymol, and therefore the permeability of the cytoplasmic membrane increased and release of K+ and ATP was carried out (92, 93). Furthermore, its integration with the polar head-group placed in the lipid bilayer leads to cell membrane alteration (94). It has been also noted that the citrate metabolic pathway and the enzymes involved in ATP synthesis would be affected by thymol (95).

Carvacrol is another component that has antimicrobial activity. It was supposed that fatty acid profiles and the structure of the cell membrane are changed by carvacrol. Moreover, it was reported that carvacrol could influence proton motive force and the synthesis of flagellin and thus reduce bacterial motility (3, 93). Furthermore, it was indicated that membrane-bound ATPase activity of *L. monocytogenes* would be inhibited by components such as carvacrol, eugenol, and cinnamaldehyde (96).

Awaisheh et al. studied the anti-*Listeria* activity of fir or qysoom EOs alone (1% v/w) or in combination (0.5% each) in beef-luncheon meat products inoculated with *L. monocytogenes* and stored at 4°C for 14 days. They figured out that at the end of storage, for samples with low contamination (3 log CFU/g), fir EO, qysoom EO, and their mixture had ~6.37, 6.04, and 5.53 log CFU/g of *L. monocytogenes*, respectively, compared to 6.90 log CFU/g of the control, while in the samples with a high contamination level (6 log CFU/g), bacterial counts reached 8.43, 8.88, and 6.75 log CFU/g for fir EO, and qysoom EO, and their mixture, respectively, compared to 9.90 log CFU/g of the control. They indicated that the combination of fir and qysoom exerts good anti-*Listeria* activity. It has been reported that α -and β-pinene, 1,8-cineol, and borneol that are present in fir and qysoom participate in the antimicrobial activities of these EOs (97).

In a study carried out by Carramiñana et al., the effect of savory (*Satureja montana)* EO [0.25, 0.5, 1, and 2.5 μl/g (v/w)] against inoculated *L. monocytogenes* serovar 4b (10^4^ CFU/g) in minced pork meat stored at 4°C for up to 7 days was investigated. Furthermore, they utilized thyme (*T. vulgaris* F) and rosemary (*R. officinalis*) as reference ingredients. They found that just *S. montana* and *T. vulgaris* F EOs efficiently inhibited the growth of *L. monocytogenes* in pork, while *R. officinalis* had no remarkable antimicrobial effect. They indicated that the low antimicrobial effect of *R. officinalis* EO could be attributed to the absence of carvacrol and thymol in this EO (92). Similarly, Bukvički et al. (98) studied the effect of *Satureja horvatii* EO (0.16–20 mg/ml) on *L. monocytogenes* (10^7^ CFU/ml) in pork meat medium stored at 4 and 25°C for 3 days. They also indicated that two concentrations of 10 and 20 mg/ml *S. horvatii* EO led to a 100 inhibition of *L. monocytogenes* regardless of the incubation temperature, while no inhibition was observed at the lowest concentrations of *S. horvatii* EO (0.16 and 0.32 mg/ml). GC-MS analysis of *S. horvatii* EO showed that the main components of this EO included *p*-cymene (33.14%), thymol (26.11%), thymol methyl ether (15.08%), γ-terpinene (4.05%), α-pinene (4.26%), and α-terpinene (4.02%) (98). It has been reported that the high antimicrobial potential of *Satureja* oil is related to the considerable amount of oxygenated monoterpenes thymol and thymol methyl ether compounds (99). Selected publications on the major compounds of various EOs and their anti-*Listeria* activity in meat and poultry products are summarized in Table 1.

**Table 1:** Major compounds of essential oils utilized in meat and poultry products against *Listeria monocytogenes*.

**Meat product**	**Type of essential oil**	**Essential oil concentration**	**The mode of essential oils applications**	**Main components**	**Outcome**	**References**
Minced beef meat	*Thymus capitata*	0.01, 0.05, 0.25, and 1.25% (v/w)	Addition of essential oil solution to minced beef meat	Carvacrol (88.98%), Linalol (1.57%), Terpinen-4-ol (1.41%), *p*-Cymene (1.14%), Caryophyllene epoxide (1.08%)	Due to high amount of carvacrol, *T. capitata* essential oil showed high antioxidant and antimicrobial activities. Application of 0.25 or 1% (v/w) of *T. capitata* essential oil along with low temperature storage can decrease potential contamination of *L. monocytogenes*.	(18)
Minced beef meat	*Ceratonia siliqua*	0.1, 0.2, and 0.4 mg/ml	Addition of essential oil solution to minced beef meat	Nonadecane (21.68%), Heneicosane (10.04%), Naphthalene (9.08%), 1,2-Benzenedicarboxylic acid dibutylester (8.88%), Heptadecane (6.56%), Hexadecanoic acid (5.83%), Octadecanoic acid (4.97%), 1,2-Benzenedicarboxylic acid (3.81%), Phenyl ethyl tiglate (2.76%), Eicosene (2.34%), Farnesol 3 (1.32%), Camphor (1.19%), Nerolidol (1.09%), and n-Eicosane (1.04%).	The concentration of 2.5 and 5 mg/ml (2 MIC) had bacteriostatic activity (MIC) while the 7.5 mg/mL exhibited a bactericidal activity (MBC). Antimicrobial activity of *C. siliqua* essential oil in contaminated minced beef meat (2 × 10^2^ CFU/g of *L. monocytogenes*) that stored at 7°C for 10 days was evident and by increasing essential oil concentration, a gradual decrease in *L. monocytogenes* count was observed. 0.1, 0.2, and 0.4 mg/mL of *C. siliqua* essential oil resulted a 2-log decrease in bacterial population after 6,4, and 2 days of refrigerated storage.	(13)
Ground beef meat	*Zataria multiflora* Boiss	0.3, 0.5, 1, and 2%	Addition of essential oil solution to ground beef meat	Thymol (29.2%), carvacrol (19.64%), burneol (6.62%), thymol methyl ether (6.55%), and o-isopropyltoluene (5.34%)	Treatments with 0.5, 1, and 2% of Zataria multiflora essential oil resulted to a significant decrease in *L. monocytogenes* population during 9 days of storage at 7°C.	(91)
Ground beef	*Melaleuca alternifolia* (tea tree)	1.5% v/w	Addition of essential oil solution to ground beef	Terpinen-4-ol (43.1%), γ-Terpinene (22.8%), α-Terpinene (9.3%), α-Terpineol (5.2%), Terpinolene (3.5%), and α-Pinene (3.0%)	The values of 0.10 μL/g and 0.15 μL/mL were obtained for MIC and MBC, respectively. Based on the inoculation volum of *L. monocytogenes* (1.5 × 108 CFU/mL, 4.6 × 104 CFU/mL, 9.2 × 103 CFU/mL, and 1.2 × 102 CFU/mL), various result was obtained. High counts of *L. monocytogenes* was observed in the control samples at all concentration during storage (14 days at 4°C). No growth of *L. monocytogenes* was observed after the first 20 min of storage in the essential oil- treated samples with initial concentrations of 1.2 × 10^2^ CFU/mL and 9.2 × 10^3^ CFU/mL, while, a reduction of 2.41 log CFU/mL was occurred in the sample with initial counts of 4.6 × 10^4^ CFU/mL. *M. alternifolia* essential oil was not significantly effective in the sample wit high initial inoculation level (1.5 × 10^8^ CFU/mL).	(100)
Sous vide cook-chill beef	*Thymus vulgaris* L. and *Rosmarinus officinalis* L.	Thyme; 3.9 μl/mL Rosemary; 62.5 μl/mL	Essential oil was added directly on meat	*Thymus vulgaris* L.: Linalool (18.18%), thymol (7.48%), limonene (6.49%), endo-borneol (5.86%) and terpinen-4-ol (5.66%).	The sample with thyme essential oils and control sample had similar count of *L. monocytogenes*. On the other hand, a 2 log CFU/g reduction was occurred in the rosemary-treated samples stored at 2 and 8°C.	(74)
				*Rosmarinus officinalis* L.: Eucalyptol (13.05%), camphor (8.93%), verbenone (8.58%), endo-borneol (7.87%) and α-pinene (6.78%)	It is essential to provide adequate chilling storage to assure the safety of the sous vide cook-chill beef in terms of *L. monocytogenes*.	
Bovine ground meat	*Syzygium aromaticum* (clove) and *Cymbopogon citratus* (DC.) Stapf (lemongrass)	1.56, 3.125, and 6.25% (w/v)	Addition of essential oil solution to bovine ground meat	Clove: eugenol (89.80%), trans-caryophyllene (5.88%) and α-humulene (2.30%) Lemongrass: geranial (42.90%), neral (30.90%) and 2-undecanone (4.1296%)	The value of The MIC value of 56% was obtained for both essential oils. The population of bacteria significantly affected in the contaminated meat sample (106 CFU/g of *L. monocytogenes*) with clove and lemongrass essential oils during 3 days incubation at 5°C. The most remarkable reductions were occurred at higher concentrations of both tested essential oils, whereas at concentrations of 3.125 and 6.25% (w/v) no bacteria was detected after the second day of storage.	(101)
Wine marinated beef	*Juniperus communis* and *Satureja montana*	*J. communis* essential oil (0.25%)*S. montana* (0.125%) and their combination.	Marination	*J. communis*: α-pinene (47.8%), sabinene (11.0%), β-pinene (8.5%), and limonene (5.8%) *S. montana*: carvacrol (30.7%), thymol (18.0%), para-cymene (15.6%), borneol (5.9%), and γ-terpinene (5.5%).	Basic red wine marinades and the ones with essential oil or mixture of essential oils significantly reduced *L. monocytogenes* population in comparison with saline control sample during 15 days at 4°C. The marinade containing mixture of tested essential oil exhibited the most pronounced effect.	(7)
Minced beef meat	*Citrus limon* (lemon)	0.06 and 0.312 mg/g (2MIC and 3MIC, respectively)	Addition of essential oil solution to minced beef meat	β-Pinene (25.44%), Limonene (39.74%), Linalool (2.16%), α-Terpineol (7.30%), linalyl acetate(3.01%), Acétate geranyl (3.03%), Nerolidol (6.91%), Acetate neryl (1.74%), and Farnesol (4.28%).	Untreated samples had higher counts of bacteria over the storage period, while the addition of *C. limon* essential oil at 2 and 3 MIC had inhibitory effect on *L. monocytogenes* during 10 days storage at 4°C. The application of C. limon essential oil at a 0.06 and 0.312 mg/g may potentially considered as new strategies in controlling growth of pathogenic bacteria, especially *L. monocytogenes* during storage of minced beef meat at 4°C.	(102)
Turkey meat	*Zataria Multiflora* Boiss and *Bunium persicum* Boiss	0.5 and 1%	In chitosan coating nanoemulsion	*Zataria Multiflora* Boiss: carvacrol (51.55%), thymol (25.49%), ρ-cymene (5.23%), and γ-terpinene (4.44%) *Bunium persicum* Boiss: cumic aldehyde (38.39%), ρ-cymene (18.36%), and 2-Caren-10-al (13.26%).	Nanoemulsions of *Zataria Multiflora* Boiss had the MIC and MBC values of 0.25 and 0.5 mg/mL, respectively, while the MIC and MBC values of nanoemulsions *Bunium persicum* Boiss were 1 and 2 mg/mL.	(31)
Chicken breast filets	Ginger (*Zingiber officinale*)	Nanoemulsion-based edible sodium caseinate with ginger essential oils (3 and 6% wt)	In nanoemulsion-based edible sodium caseinate coating	a-zingiberene (24.96%) b-sesquiphellandrene (12.74%), sesquisabinene hydrate (6.19%), camphene (5.90%), zingiberenol (4.26%), (E)-citral (3.93%), sabinene (3.75%), (E)-farnesene (3.73%), and italicene (3.21%)	Nanoemulsion based edible coatings with 6% of ginger essential oils nanoemulsion led to remarkable reduction in *L. monocytogenes* in refrigerated chicken filets during 12 days.	(103)
Chicken meat filets	Rosemary and cinnamon essential oils	Sodium alginate active coating solutions containing, cinnamon and rosemary essential oils (5 mg/ml) and nisin (2000 IU/ml) individually or in combination.	In sodium alginate coating	Cinnamon: (E)-cinnamaldehyde (83.47%), α-copaene (2.57%), and α-muurolene (1.97%) Rosemary: Camphor (23.17%), α-Pinene (18.56%), and 1,8-Cineole (11.89%)	Application of tested essential oil and nisin was effective in *controlling L. monocytogenes* and the strongest effect was observed in samples coated with alginate solution containing both cinnamon and rosemary essential oil had highest effect, indicating synergist effect of cinnamon and rosemary essential oil.	(80)
Chicken meatballs	*Ziziphora clinopodioides*	0.1, 0.2, and 0.3% v/w	Addition of essential oil solution to chicken meatballs	Carvacrol (65.22%), thymol (19.51%), p-cymene (4.86%), and γ -terpinene (4.63%).	Contaminated samples (10^5^ CFU/g of *L. monocytogenes*) with *Z. clinopodioides* essential oil had better microbial properties compared to the control during 12 days storage 4°C. In control sample although L. monocytogenes did not growth, however it survived in the refrigerated condition. While, the counts of *L. monocytogenes* reached to 2.66, 2.25, and 2.01 log CFU/g in the samples containing 0.1, 0.2, and 0.3% *Z. clinopodioides* essential oil, respectively. They concluded that *Z. clinopodioides* essential oil can be utilized as a natural substance to control L. monocytogenes in raw chicken meatball that stored at 4°C.	(19)
Sausages	Thyme essential oil	0.1%	In formula incorporation	Thymole(38.2%), p-cymene (25.4%) and terpineol with g terpirene (16.2%)	*L. monocytogenes* was inhibited by addition of thyme essential oil. The main antimicrobial component of thyme essential oil related to thymol that disturbs cell membrane and inhibits the ATPase activity of *L. monocytogenes*.	(104)
Italian mortadella	*Thymus vulgaris* L. and *Rosmarinus officinalis* L	0.025 and 0.05%	In formula incorporation	*Thymus vulgaris* L.: Thymol (45.9%), p-cymene (26.59%), linalool (4.96%) *Rosmarinus officinalis* L: α-Pinene (23.98%), camphor (22.62%), eucalyptol (18.76%), camphene (8.83%), β-pinene (5.61%)	Mixture of rosemary and thyme had anti-listeria activity in Italian mortadella. There was significant differences among control and treated samples in *L. monocytogenes*, at the end of storage (4°C, 30 d). In comparison to control samples, *L. monocytogenes* charges were almost lower of 2.29 and 2.79 log CFU/g in samples containing 0.025 and 0.05% essential oils, respectively.	(12)
Dry Fermented Sausages	*Juniperus communis* L.	0.01, 0.05, and 0.10 μL/g	In formula incorporation	β-myrcene (14.12%), sabinene (9.51%), d,l-limonene (8.36%), 4-terpineol (6.88%), α-amorphene (5.43%), β-pinene (5.39%), caryophyllene (3.94%), p-cymene (3.92%), germacrene D (3.81%),	No foodborne pathogens (*Escherichia coli, Listeria monocytogene*s, *Salmonella* spp. and sulfite-reducing clostridia) were observed in any sample throughout the storage period (225 days). The sample with 0.10 μL/g of *Juniperus communis* L. exhibited untypical flavor. *Juniperus communis* L. essential oil can be considered as partial replacement for sodium nitrite in dry fermented sausages.	(105)
Dry cured sausages (Portuguese chouriço de vinho)	Bay, garlic, nutmeg, oregano, rosemary, thyme	0.005 and 0.05%	In formula incorporation	Bay: Eucaliptol (58.20%), α-terpinenyl acetate (19.19), b-phellandrene (5.01) Garlic: Diallyl trisulfide (33.82%), diallyl disulfide (18.86%), diallyl tetrasulphide (10.97%), methyl allyl trisulfide (9.04%), diallyl sulfide (8.36%) Nutmeg: Myristicin (43.35%), sabinene (23.28%) Oregano: Thymol (93.34%), γ-Terpinene (1.29%) Rosemary: Camphor (22.4%), eucaliptol (13.24%), a-pinene (10.84%) Thyme: Thymol (93.94%) cis-Ocimene (1.29%)	Microbial counts of the tested pathogens was reduced by the addition of essential oils and by increasing concentration, higher antimicrobial effect was observed. However, due to sensory limitations, application of high concentrations was not practical. *L. monocytogenes* was not detected in the sample with 0.005% of oregano, rosemary and nutmeg, after 12 days of drying. Contribution of essential oil addition (0.005%) for remarkable reduction of pathogen's counts and for a shorter period to obtain the not detectable level, making the industry to decrease the drying period and increasing yield production.	(106)
Tuscan sausage	Bay leaf	0.05% and 0.1%	In formula incorporation	1.8-Cineole (35.50%), linalool (14.10%), α-terpinyl acetate (9.65%), sabinene (9.45%)	The count of psychrotrophic microorganisms such as *L. monocytogenes* were significantly lower (*P* < 0.05) in the treated samples than in the control during 14 days storage at 7°C. The count of psychrotrophic microorganisms reached to 7 log CFU/g on day 8 for the sample treated with 0.05% of essential oil, and on day 10 for the sample containing 0.1%, indicating a 2-day increase in shelf life with 0.1 g/100 g of bay leaf essential oil.	(107)
Sausage model	Chinese cinnamon and cinnamon bark	0.025 and 0.05 v/w mixed essential oil	Emulsified microbeads added into sausages formulation	Chinese cinnamon: Trans-Cinnamaldehyde (87.58%) and cinnamyl acetate (7.53%) Cinnamon bark: Transcinnamaldehyde (40.71%), cinnamyl acetate (14.25%), β- phellandrene (9.02%), and β -caryophyllene (7.41%)	Anti-listerial effects of essential oils, nisin, nitrite and organic acid salts in a sausage was studied during 7 days storage at 4°C. Application of 0.025 and 0.05% essential oil in combination with nitrite (100 ppm), organic acid salts (1.55%), and nisin (12.5 ppm), led to a 1.5 and 2.6 log CFU/g reduction in *L. monocytogenes* population, respectively in comparison with control at day 7 of storage.	(108)
Pork Sausages	Lemongrass	2%	Poly lactic acid films	Not detected	The population of inoculated *L. monocytogenes* in the sausage samples wrapped with the poly lactic acid films containing 2% lemongrass was 1.47 Log CFU/g lower than the control samples during 12 days storage at 4°C.	(109)
Turkey ham	Rosemary	1%	Addition of rosemary solution directly to the diced turkey ham	Not detected	The positive control and the sample treated with rosemary were similar in *L. monocytogenes* counts during 63 days at 4°C.	(110)
Bologna	Oregano	1 or 2%	Chitosan films with oregano essential oil	Not detected	Chitosan films alone decreased *L. monocytogenes* counts, by value of 2 logs CFU/g during 5 days storage at 4°C, while addition of 1 and 2% oregano essential oil resulted in a reduction of 3.6 and 4 logs CFU/g in population of *L. monocytogenes*, respectively, indicating antibacterial potentiality of oregano essential oil.	(111)
Ham Slices	Oregano	1% (v/v)	In Na-alginate edible films	Not detected	Control treatment had slightly higher counts of *L. monocytogenes*, compared to the sample treated with edible film containing oregano essential oil during 40 days at 4, 8 and 12°C. A 1.5 log CFU/g reduction in *L. monocytogenes* population was observed at the end of storage at 8 and 12°C, while at 4°C, a reduction 2.5 log CFU/g was occurred.	(85)
Mortadella-type sausages	*Zataria multiflora* Boiss	0.5 and 1%	Chitosan films containing essential oil	Not detected	The highest count of *L. monocytogenes* was observed in control sample during 6 days at 4°C. Essential oil addition inhibited *L. monocytogenes* growth and anti-listeria activity of chitosan film significantly changed by increasing essential oils concentration from 0.5 to 1%.	(112)
Chicken frankfurters	*Thymus daenensis* Celak, *Thymbra spicata* L. and *Satureja bachtiarica* Bunge	1% (v/w)	Spraying on the surface	Not detected	Control sample had significantly higher *L. monocytogenes* population compared to the samples treated with essential oils during 14 days of storage at 4°C.The highest decrease in bacterial population was observed in the sample treated with *Thymus daenensis* essential oil.	(113)

## Limitations and Future Trends of EO Application in Meat and Poultry Products

The use of EOs as additives and preservatives has a long history, and the use of these compounds along with their toxicity has been considered. One of the substantial aspects of EOs is that they are generally low-risk products. It has been reported by various studies that EOs and their chemical components possess a range of 1 to 20-g/kg body weight for LD50 values, but some exceptions are also observed (114).

Most EOs are nontoxic to mammals and fish, but they are good as pesticides (115). Generally, the chemical compounds that are present in EOs have no remarkable risk that is derived from oral intake. Therefore, the toxicity of EOs is principally related to the compounds that exist in the EO. It has been reported that most of the EO constituents, even at high levels, exhibited no carcinogenic effects (116).

EOs are widely utilized in various products including detergents, creams, lotions, perfumes, and soaps and different food products such as beverages, baked foods, puddings, and meat products. They are well known for their antimicrobial properties and can be applied as antiseptic agents (117, 118). Therefore, EOs can be considered as an alternative for chemical preservatives in food products such as meat and meat products (119).

The efficiency of various EOs has been reported in the control and inhibition of *L. monocytogenes* in meat and poultry products; however, it seems that the interaction of EOs with various food constituents might decrease their antimicrobial properties (3, 53, 73, 120). The interaction of the phenolic compounds of EO with proteins and surrounding EO hydrophobic constituents with fat seem to limit their availability to microorganisms' target size, and therefore, their antimicrobial properties decrease (53). Furthermore, it has been indicated that antimicrobial activities of EOs increase by reduction of oxygen level and pH. The hydrophobicity of EOs would increase by a decrease in pH, making it easy for them to dissolve the cell membrane and interact with target sites of the microorganism (3, 120, 121). High concentration of EOs is required for acceptable antimicrobial activity. Consequently, due to the intense aroma of EOs, their application in higher amounts could result in sensory defects (60, 119).

In order to rectify this shortcoming, various approaches have been suggested. Incorporation of EOs to edible films and coatings, microencapsulation or nanoencapsulation of EOs, use of EO mixtures, and concomitant use of EOs with other preservation methods such as low temperature, new packaging methods, thermal and nonthermal processes can be named as examples of these strategies (3, 31, 119, 122, 123). It has been reported by Khaleque et al. that clove EO (10%) inactivated *L. monocytogenes* in ground beef meat; however, no consumer liked 10% clove EO-supplemented cutlets due to the strong flavor. Therefore, there is a limitation of using spices with a strong flavor like clove. They suggested that a combination of other preservatives such as acid or salt with clove EO and proper storage condition can be useful in reducing the unfavorable sensory effect of clove EO (4).

For example, Upadhyay et al. figured out that combinations of β-resorcylic acid (1.5%), carvacrol (0.75%), and *trans*-cinnamaldehyde (0.75%) with hydrogen peroxide (0.1%) inhibited the growth of *L. monocytogenes* more effectively than did individual tested antimicrobial compounds or hydrogen peroxide (71).

In addition, Noori et al. studied the antimicrobial properties of nanoemulsion-based edible sodium caseinate coating containing ginger EO (3 and 6% wt.) on chicken breast filets. They found that nanoemulsion-based edible coating containing 6% of ginger EO nanoemulsion significantly decreased total aerobic psychrophilic bacteria during 12 days of storage. It seems that nanoemulsion formation results in a reduction in EO droplet size and therefore there is faster penetration of the antimicrobial compounds into the bacterial cell. Hence, this increased antimicrobial activity of nanoemulsions, in comparison with conventional emulsions, allows the application of lower concentrations of EOs in food and active coating or packaging (103). Furthermore, it has been reported that chitosan-loaded nanoemulsion containing *Z. multiflora* EO increased reduction of *L. monocytogenes* in turkey meat in comparison to control during 18 days of storage at 4°C (31). Moreover, the effect of applying VP and MAP conditions with or without the bay EO on controlling *L. monocytogenes* in ground chicken breast was studied by Irkin et al. They found that VP and MAP efficiency against *L. monocytogenes* can be enhanced by the addition of bay EO in chicken meat (124). Furthermore, it has been reported by Pavli et al. that the combined effect of high-pressure processing and sodium alginate edible films containing oregano EO led to more reduction of *L. monocytogenes* in a shorter time and with the lowest final levels in ham slices in comparison to the ones where high-pressure processing and edible films with oregano EO were separately utilized (85). Additionally, it has been reported by Criado et al. that there was a synergistic effect among thyme EO and irradiation on the reduction of the *Listeria innocua* population and extension of the shelf life of ground meat (30). Similarly, the combined effect of gamma (γ)-irradiation and microencapsulated oregano and cinnamon EO and nisin against *L. monocytogenes* on RTE ham was investigated by Huq et al. They found that γ-irradiation treatment and microencapsulated antimicrobials showed a synergistic antimicrobial effect on RTE meat products during storage (125).

Additionally, Cui et al. studied the synergetic antimicrobial effects of cold nitrogen plasma and lemongrass oil against *L. monocytogenes* on pork loin. They found that antibacterial activities of lemongrass oil against *L. monocytogenes* was obtained at high doses, while with exertion of cold nitrogen plasma, a lower concentration of lemongrass oil is required to achieve acceptable antimicrobial effects. Therefore, the possible adverse sensory effect of high concentrations of EO can be mitigated after synergic treatment (126). Therefore, it could be stated that combined use of EOs as well as their application with other preservative and packaging methods has a synergistic antimicrobial effect on *L. monocytogenes* in meat and meat products, and further reduction of *L. monocytogenes* occurred when EOs are associated with these preservative methods (12, 31, 71, 80, 84, 86, 103).

## Conclusion

This study has revealed that EOs could be potentially used as a replacement for chemical preservatives in meat and poultry products to mitigate or inhibit growth of *L. monocytogenes*. The antimicrobial properties of EOs are ascribed to the action of various compounds that are present in the EOs. Thymol, carvacrol, eugenol, carvone, cinnamaldehyde, limonene, α- and β-pinene, and *p*-cymene can be named as examples of major compounds of EOs that exert anti-*Listeria* activity through different mechanisms such as changing fatty acid profiles and the structure of cell membrane and increasing cell permeability as well as affecting membrane proteins and inhibition of functional properties of the cell wall. However, due to the possible negative effect of EOs, especially in high concentrations, on the organoleptic properties of meat and poultry products, the concentration of these substances utilized in meat and poultry products should be carefully taken into consideration. Furthermore, a combination of low amounts of EOs with other natural antimicrobial substances and technologies which exert synergistic antimicrobial effects could be applied to efficiently prevent growth of *L. monocytogenes* in meat and poultry products. Incorporation of EOs to edible films and coatings, microencapsulation or nanoencapsulation of EOs, use EO mixtures, and application of EOs with new packaging methods and emerging technology such as high hydrostatic pressure, irradiation, high-intensity pulsed electric field, and cold plasma can be more efficient in inhibiting *L. monocytogenes* growth and enhancing the safety and quality of meat and poultry products, which should be studied in future investigations.

## Author Contributions

This study was design by HH. The manuscript was written by MY and NK. HH critically revised the manuscript, and finally, all authors listed have approved it for publication. All authors contributed to the article and approved the submitted version.

## Conflict of Interest

The authors declare that the research was conducted in the absence of any commercial or financial relationships that could be construed as a potential conflict of interest.
